# Draft Genome Sequences of *Algoriphagus* sp. Strain PAP.12 and *Roseivirga* sp. Strain PAP.19, Isolated from Marine Samples from Papua, Indonesia

**DOI:** 10.1128/mra.01264-22

**Published:** 2023-03-16

**Authors:** Riyanti, Celine M. Zumkeller, Marius Spohn, Sanja Mihajlovic, Oliver Schwengers, Alexander Goesmann, Nur A. Choironi, Till F. Schäberle, Harwoko Harwoko

**Affiliations:** a Faculty of Fisheries and Marine Science, Jenderal Soedirman University, Purwokerto, Indonesia; b Branch for Bioresources, Fraunhofer Institute for Molecular Biology and Applied Ecology, Giessen, Germany; c Bioinformatics and Systems Biology, Justus Liebig University Giessen, Giessen, Germany; d German Center for Infection Research, Partner Site Giessen-Marburg-Langen, Giessen, Germany; e Department of Pharmacy, Faculty of Health Sciences, Jenderal Soedirman University, Purwokerto, Indonesia; f Institute for Insect Biotechnology, Justus Liebig University Giessen, Giessen, Germany; Montana State University

## Abstract

*Algoriphagus* sp. strain PAP.12 (EXT111900) and *Roseivirga* sp. strain PAP.19 (EXT111901) were isolated from marine samples. Here, we report their draft genome sequences, 5.032 Mbp and 4.583 Mbp in size, respectively, and rate their specialized metabolite production capacity. Taxonomic ranks established by genome-based analysis indicate that *Algoriphagus* sp. strain PAP.12 represents a candidate new species.

## ANNOUNCEMENT

The phylum *Bacteroidetes* is a proliferating but underexplored bioresource for natural product discovery (1, 2). The bacteria colonize diverse habitats and are among the most abundant groups of bacteria within marine ecosystems (3). In an effort to access new *Bacteroidetes* strains, strains PAP.12 and PAP.19 were isolated from Cenderawasih Bay National Park (Papua, Indonesia). The strains were deposited in the Fraunhofer strain collection (4) under the identifiers EXT111900 and EXT111901.

EXT111900 was retrieved from the upper layer (5 to 10 cm) of marine sediment (1°47′45.6″S, 134°25′12.0″E). EXT111901 was isolated from a sponge (1°48′03.6″S, 134°06′03.6″E) by cutting using a diving knife while latex gloves were worn. The samples were put into sterile bags, brought to the surface to be kept at ambient seawater temperature, and processed within 2 hours after sampling, as follows. Sponge samples were cut into small pieces using a sterile scalpel, and all samples were rinsed with sterile seawater before being plated on artificial seawater (1.5% agar, 0.01% KBr, 2.3% NaCl, 1.1% MgCl_2_·6H_2_O, 0.1% CaCl_2_·2H_2_O, 0.1% KCl, 0.004% SrCl_2_·6 H_2_O, 0.4% Na_2_SO_4_, 0.02% NaHCO_3_, and 0.003% H_3_BO_3_ in H_2_O) with Escherichia coli prey and cycloheximide. Bacterial colonies were isolated, and sequencing samples were prepared by growing the strains aerobically for 24 h at 30°C in marine broth (product number CP73.1; Carl Roth GmbH). Cell pellets were resuspended in buffer ATL (Qiagen) containing RNase A. ZR BashingBead lysis tubes (Zymo Research) were used for cell disruption. DNA was isolated using QIAmp 96 DNA QIAcube high-throughput (HT) kits with the addition of proteinase K (Qiagen). Libraries for short-read sequencing were prepared using the Illumina DNA preparation tagmentation kit with 500 ng DNA as input and 5 cycles of indexing PCR. Library quality was evaluated (Agilent 2100 Bioanalyzer), and libraries were sequenced on an Illumina NovaSeq system using a NovaSeq 6000 SP v1 sequencing kit with 2 × 150 bp read length and a depth of 4.0 to 5.0 million reads per sample. For sequence processing and analysis, software tools were run with default settings unless otherwise stated. The sequencing was demultiplexed (Illumina bcl2fastq v2.19.0.316), quality checked (Fastp v0.20.1 [5], with additional parameters –detect_adapter_for_pe, –cut_by_quality5, –cut_by_quality3, –low_complexity_filter, –length_required 21, and –correction), and visualized (MultiQC v1.7 [6]). Paired-end reads were quality filtered (Fastp v0.20.1 [5]), assembled (Unicycler v0.4.8 [7]), and quality checked (CheckM v1.0.18 [8]), and genomes were annotated using Bakta v1.5.1 (9). The genome of strain EXT111900 consists of 5,032,044 bp in 101 contigs (coverage, 405×; *N*_50_, 235,524 bp), with a GC content of 39.4%, revealing completeness of 99.81% and contamination of 0.19%. The genome contains 4,286 protein-coding genes, 41 tRNAs, 1 transfer-messenger RNA (tmRNA), 3 rRNAs, and 3 noncoding RNAs (ncRNAs). The genome of strain EXT111901 consists of 4,582,827 bp in 6 contigs (coverage, 362×; *N*_50_, 3,058,286 bp), with a GC content of 42.1%, revealing completeness of 99.81% and no contamination. The genome contains 3,944 protein-coding genes, 47 tRNAs, 1 tmRNA, 6 rRNAs, and 3 ncRNAs.

Taxonomic ranks were established by using the Type Strain Genome Server (TYGS) (10) and autoMLST (11) and by determining the average nucleotide identity (ANI) (12). Roseivirga pacifica DY53 (13) represents the closest related type strain of EXT111901. Digital DNA-DNA hybridization (dDDH) values 86.6% (d0), 70.8% (d4) and 86.7% (d6) and an ANI of 96.65%, all exceeding the species delineation threshold, support affiliation of EXT111901 with the species R. pacifica. The closest related type strain for EXT111900 is Algoriphagus zhangzhouensis DSM 25035 (14). The dDDH values are 71.8% (d0), 35.8% (d4), and 62.9% (d6), and the ANI is 88.84%. Accordingly, EXT111900 possibly represents a candidate new *Algoriphagus* species.

**FIG 1:**
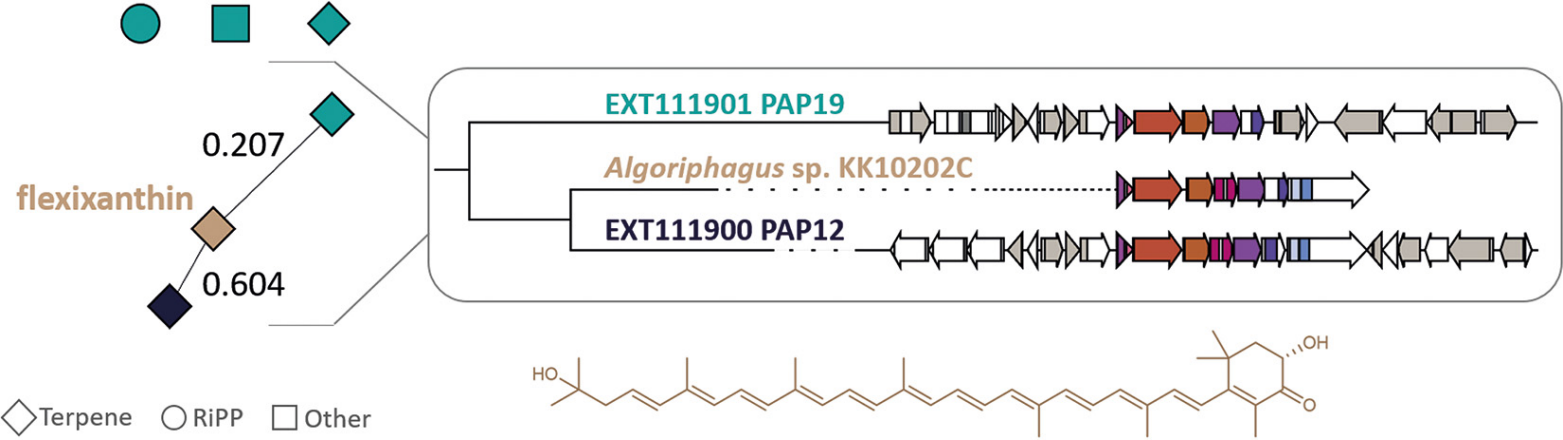
BiG-SCAPE network (left) of strains EXT111901 (turquoise) and EXT111900 (dark blue). BGCs sharing similarity with the flexixanthin MIBiG cluster were aligned (right). EXT111901 carries two terpene BGCs, a ribosomally synthesized and posttranslationally modified peptide (RiPP) BGC, and a homoserine lactone (Other) BGC. Terpene BGCs of EXT111901 and EXT111900 each cluster with the flexixanthin BGC0000650 of *Algoriphagus* sp. strain KK10202C (similarity index values of 0.207 and 0.604, respectively). A difference is the fusion-type lycopene β-cyclase (pink open reading frame [ORF]) present in EXT111900 and absent in EXT111901. The structure of flexixanthin is depicted at the bottom.

Prediction of biosynthetic gene clusters (BGCs) was performed using antiSMASH v6.0 (15). Their grouping into gene cluster families (GCFs) together with the Minimum Information about a Biosynthetic Gene cluster (MIBiG) (16) reference clusters using BiG-SCAPE (17) with a similarity index cutoff value of 0.6 allowed BGC similarity determinations. EXT111900 carries one BGC clustering with BGC0000650, encoding the carotenoid flexixanthin (18). Although less similar, the same match was detected for EXT111901 (Fig. 1). A difference is the presence of a lycopene β-cyclase in EXT111900 and its absence in EXT111901.

### Data availability.

The whole-genome shotgun projects have been deposited in GenBank under BioProject accession number PRJNA904539 with BioSample accession numbers SAMN31845353 (EXT111900) and SAMN31845354 (EXT111901). Raw sequencing reads can be obtained from the Sequence Read Archive (SRA) (SRA accession number SRP421610). The draft genome sequences have been deposited in GenBank under the accession numbers JAPPSQ000000000 (EXT111900) and JAPPSP000000000 (EXT111901).
